# Valuation of Normal Range of Ankle Systolic Blood Pressure in Subjects with Normal Arm Systolic Blood Pressure

**DOI:** 10.1371/journal.pone.0122248

**Published:** 2015-06-08

**Authors:** Yi Gong, Kai-wu Cao, Jin-song Xu, Ju-xiang Li, Kui Hong, Xiao-shu Cheng, Hai Su

**Affiliations:** Department of Cardiology, Second Affiliated Hospital of Nanchang University, Nanchang, Jiangxi, People’s Republic of China; The University of Tokyo, JAPAN

## Abstract

**Subject:**

This study aimed to establish a normal range for ankle systolic blood pressure (SBP).

**Methods:**

A total of 948 subjects who had normal brachial SBP (90-139 mmHg) at investigation were enrolled. Supine BP of four limbs was simultaneously measured using four automatic BP measurement devices. The ankle-arm difference (An-a) on SBP of both sides was calculated. Two methods were used for establishing normal range of ankle SBP: the 99% method was decided on the 99% reference range of actual ankle BP, and the An-a method was the sum of An-a and the low or up limits of normal arm SBP (90–139mmHg).

**Results:**

Whether in the right or left side, the ankle SBP was significantly higher than the arm SBP (right: 137.1±16.9 vs 119.7±11.4 mmHg, P<0.05). Based on the 99% method, the normal range of ankle SBP was 94~181 mmHg for the total population, 84~166 mmHg for the young (18–44 y), 107~176 mmHg for the middle-aged(45–59 y) and 113~179 mmHg for the elderly (≥60y) group. As the An-a on SBP was 13mmHg in the young group and 20mmHg in both middle-aged and elderly groups, the normal range of ankle SBP on the An-a method was 103–153 mmHg for young and 110–160 mmHg for middle-elderly subjects.

**Conclusion:**

A primary reference for normal ankle SBP was suggested as 100-165 mmHg in the young and 110-170 mmHg in the middle-elderly subjects.

## Introduction

Ankle blood pressure (BP) is used more frequently in clinical practice as it could be easily taken using an electronic BP device [1–4]. Previously, ankle BP was mainly used for calculating ankle-brachial index (ABI) [5, 6]. Although ankle BP has been demonstrated a predictor for subclinical atherosclerosis, cardio-cerebrovascular morbidity and mortality [2–4], the understanding on ankle BP per se is insufficient at present. For example, the normal range of ankle BP is still uncertain, although one study considered >175 mmHg as elevated ankle systolic BP (SBP) [3]. Generally, arm SBP is used as the reference for identifying the abnormality of ankle SBP in most studies [7, 8].

The number of patients with occlusive subclavian and brachial artery disease is rising. In some patients, arm BP could not be taken or could not correctly reflect their real BP [9–13]. In these cases, taking ankle BP is an alternative way. Meanwhile, ankle BP is also often measured in operation. Increasing use of ankle BP needs a reference for normal range of ankle BP. This study evaluated the ankle systolic BP (SBP) in the subjects with normal arm SBP in order to provide a reference for normal ankle SBP.

## Subjects and Methods

### Subjects

This study was approved by the Ethics Committee of the Second Affiliated Hospital of Nanchang University. After explaining the value of examination, all patients provided verbal informed consent as the BP measurement of four limbs is a non-invasive clinical examination. The results were recorded in medical record by a research assistant nurse for following up. The ethics committee approved this consent procedure.

From May to November of 2013, 948 subjects (47.9±18.5y) with normal SBP (90–139 mmHg), physical check and electrocardiogram at investigation were included. The exclusion criteria were hypertensive history, diabetes, arrhythmia, acute myocardial infarction, aortic coarctation, congenital heart disease, heart failure, hemiplegia, pulseless disease and the history of trans-radial coronary intervention.

According to WHO standards, the young (334 subjects, 50.7% males, 18-44y, mean 26.1±7.5y,), the middle-aged (295 subjects, 44.4% males; 45-59y, mean 51.5±5.5y) and elderly (319 subjects, 48.9% males; ≥60y, mean 67.4±6.2y) were created. The body mass index (BMI), smoking percentage and serum total cholesterol (TC) levels were higher in the middle-aged and elderly groups than, but levels of triglyceride and fasting glucose were similar to the young group (Table 1).

**Table 1:** The general information of the total and three age groups.

	N	Male	Age	BMI	Smokingn	TC	TG	FPG
		(%)	(y)	(kg/m2)	(%)	(mmol/L)	(mmol/L)	(mmol/L)
Young	334	50.7%	26.1±7.5	20.2±2.7	17 (5.1)	4.08±0.79	1.57±1.21	4.66±0.98
Middle-aged	295	44.4%	51.5±5.5^**#**^	25.7±2.4^**#**^	51 (17.3)^**#**^	4.68±0.85^**#**^	1.55±1.11	4.54±0.96
Elderly	319	48.9%	67.4±6.2^**#**^ ^*^	23.1±2.8^**#**^ ^*^	27 (8.5)^**#**^ ^*^	5.15±1.28^**#**^ ^*^	1.48±1.36	4.63±0.99
Total	948	48.2%	47.9±18.5	22.9±3.5	96 (10.0)	4.63±1.09	1.53±1.23	4.61±0.98

BMI: body mass index; TC: total cholesterol; TG: triglyceride; FPG: fasting glucose.

^**#**^ compared with Young P<0.05.

^*^compared with Middle-aged P<0.05.

### Methods

BP was measured in an air-conditioned room at a temperature of 22–23°C. Before BP measurement, the subjects were asked to empty their bladder, bare four limbs and take a 10 min rest in supine position. The supine BP of four limbs was simultaneously measured using four automatic BP measurement devices (Omron, HEM-7112) for 4 times with a 2-min interval, and the average of the last 3 readings was recorded as the final BP.

The ankle-arm differences (An-a) on SBP in both sides were calculated, respectively. The 95% and 99% reference ranges on the actual ankle BP values were calculated. As all participants had normal arm SBP of 90-139mmHg, the low and up limits of normal ankle SBP were the sum of An-a and 90 and 139 mmHg (An-a method), for example, the sums of 90 or 139 mmHg and the An-a on SBP were used as the low and upper limits of the ankle SBP. Meanwhile, the low and up limits of normal ankle SBP were decided on the 99% reference range of actual ankle BP (99% method). Finally, the mean values of the low or up limits from both methods were used as the reference of the low or up limits for ankle SBP.

The agreement between arm and ankle SBP was evaluated by the Bland-Altman method [14]. The An-a on SBP were plotted against the mean An-a on SBP and arm SBP in three age groups. The 95% limits of agreement (LoA) were determined (95% LoA = mean difference ± 1.96 standard deviation).

### Statistical analysis

Data was created in Excel 2003 and analyzed with SPSS10.0. Continuous variables were expressed as mean ± SD. The t-test and variance (ANOVA) test were used for statistical analysis. P<0.05 was considered statistically significant.

## Results

The SBP of four limbs in three age groups are shown in Table 2. The ankle SBP was similar between two sides, although the right arm SBP was slightly higher than left arm in three age groups. As age increased, the ankle SBP of both sides increased as arm SBP did.

**Table 2:** The SBP of four limbs in the total and three age groups (mmHg).

	Right arm	Left arm	Right ankle	Left ankle
Young	112.7±10.6	111.4±10.5*	124.8±16.0	125.1±15.5
Middle-aged	120.9±10.3^**#**^	119.6±10.9^**#**^ *	141.3±13.5^**#**^	139.4±13.1^**#**^ ^**■**^
Elderly	125.8±9.1^**#**^ ^★^	125.4±9.0^**#**^ ^★^	146.0±12.7^**#**^ ^★^	144.9±12.7^**#**^ ^★^ ^**■**^
Total	119.7±11.4	118.7±11.7*	137.1±16.9	136.2±16.3^**■**^

^**#**^:compare with Young, P<0.05

^★^:compare with Middle-aged, P<0.05.

*:compare with right arm, P<0.05

^■^compare with right ankle, P<0.05.

The ankle SBP was significantly higher than the arm SBP in three age groups (Table 2).

Whether in right or left side, the An-a on SBP of the middle-aged or the elderly groups was significantly higher than the young group. But no significant difference was found between the middle-aged and elderly groups. The mean An-a on SBP of both sides was about 18 mmHg in the total population. However, this value was about 13 mmHg in the young group and 20 mmHg in the middle-elderly groups (Table 3).

**Table 3:** The An-a on SBP in the total and three age groups (mmHg).

	Right	Left
Young	12.1±12.8	13.6±12.0^*^
Middle-aged	20.4±9.0^**#**^	19.8±8.8^**#**^
Elderly	20.2±9.8^**#**^	19.5±9.6^**#**^
Total	17.4±11.4	17.6±10.7

SBP: systolic blood pressure; An-a: ankle-arm pressure difference.

^#^Compare with Young, P<0.05.

^*^Compare with right side, P<0.05.

**Fig 1:**
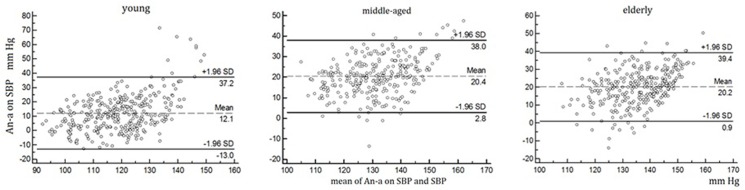
The Bland-Altman plots for SBP between right ankle and arm in three age groups. Y: the youth; M: the middle-aged; E: the elderly; SBPan: ankle SBP; SBPa: arm SBP.

The Bland-Altman plots on SBP in the right limbs are shown in the Fig 1. The mean An-a on SBP was 12.1 mmHg in the young, 20.4 mmHg in the meddle-aged and 20.2 mmHg in the elderly groups. Meanwhile, their 95% low and up limits were -13.0~37.2mmHg, 2.7~38.0 mmHg and 0.9~39.4 mmHg (Fig 1). Similar results were seen in the left limbs.

Based on the 99% method (right side), the normal ranges of ankle SBP were 93.5~180.7 mmHg for the total population; 83.5~166.1 mmHg for the young, 106.5~176.1 mmHg for the middle-aged and 113.2~178.8 mmHg for the elderly groups (Table 4).

**Table 4:** The 95, 99 reference range of SBP of right ankle in the total and three age groups (mmHg).

	95%RR	99%RR	Actual
Young	93.4~156.2	83.5~166.1	94.0~176.7
Middle-aged	114.8~167.8	106.5~176.1	113.0~185.7
Elderly	121.1~170.9	113.2~178.8	113.0~184.3
Total	104.0~170.2	93.5~180.7	94.0~185.7

RR: reference range; Actual: actual measurement values.

On the An-a method, these values were 108~158 mmHg for the total population, 103~153 mmHg for the young and 110~160 mmHg for middle-elderly groups. Finally, the averages of the low and up limits from two methods were used for establishing a reference of normal ankle SBP: 100–165 mmHg for the young and 110–170 mmHg for the middle-elderly subjects.

## Discussion

The present study showed that the ankle SBP was 18mmHg higher than arm SBP in 960 subjects with normal arm SBP. Previous studies in healthy volunteers also showed similar results, 22mmHg by Engvall et al. [7] and 17.8 mmHg by Swiet et al [8]. Furthermore, our study showed that An-a on SBP is age-dependent, 13mmHg in the young group, but 20 mmHg in both middle-aged and elderly groups.

For demonstrating the constant relationship between arm and ankle SBP, the standard of the International Protocol of the European Society of Hypertension for validation of BP devices was used in this study [15]. Based on this standard, if the percentage of the paired BP values with a difference of ≤15mmHg is over 93%, the tested BP device could be passed the validation. In this study, the percentage of SBP difference of ≤15 mmHg was 81.7% in the young, 91.2% in the middle-aged and 90.6% in the elderly group. Although these values were less than 93%, here, we must to point out that the standard of over 93% may be too strict for this study. For validation of BP devices, the measured arm BP is almost stable. However, this study evaluated the relationship of ankle SBP with arm SBP, while ankle SBP was about 18 mmHg higher than arm SBP. Therefore, using a difference of ≤20 mmHg to assess the constant relationship between ankle and arm SBP was reasonable. On this standard, the percentages of agreement between ankle and arm SBP in all three age groups were over 93%. Therefore, a constant relationship between ankle and arm SBP could be confirmed.

In this study, the normal range of ankle SBP was decided on 99% reference based on two reasons: one is that all participants have normal arm SBP, the other is to exclude some extreme deviation of SBP measurement. As a result, the normal range of ankle SBP was 94~181 mmHg for total population, 84~166 mmHg for the young, 107~176 mmHg for the middle-aged and 113~179 mmHg for the elderly groups.

Because of the constant relationship between ankle and arm SBP, An-a on SBP may be used to establish the normal range of ankle SBP. On this method, the normal ankle SBP were 108–158 mmHg for total population, 103–153 mmHg for the young, and 110–160 mmHg for both middle-aged and elderly groups.

For simple and easy purpose in clinical practice, the averages of the low and up limits from two methods were finally used as the normal range ankle SBP: 100–165 mmHg for the young and 110–170 mmHg for the middle-elderly subjects.

### Clinical implications

This study provides a primary reference for normal ankle SBP in clinical and research study.

As ankle-arm SBP difference varies with age, this age-associated variability should be taken into consideration when coarctation and leg artery stenosis is diagnosed.

### Limitation

This study was not performed in a large population with standard sampling method on epidemic research, thus there may be some bias. The normal arm SBP was decided on supine rather than sitting BP measurement, so this reference may be not completely suitable for sitting SBP. Furthermore, this study was limited to the Chinese population, so this reference may be not completely suitable in other races.

Usually, arterial compliance becomes lower in the peripheral artery than in the proximal artery. This produces pulse pressure amplification, a phenomenon of SBP increase in distal arterial site. Therefore ankle SBP is generally higher than arm SBP, especially in middle-elderly subjects. However, the Fig 1 shows negative An-a on SBP in some middle-elderly subjects, which may mean that the ankle SBP was lower than arm SBP. The reason for the negative An-a on SBP is unclear now, as no arterial imaging and simultaneous arterial wave were received. The possible mechanisms may be that those patients may have extremely higher peripheral arterial compliance or unrecognized peripheral arterial disease in low limbs.

## Conclusion

This study primarily suggests 100–165 mmHg in the young and 110–170 mmHg in the middle-elderly subjects as normal range of ankle SBP.

## Supporting Information

S1 Dataset (XLSX)
